# A Systematic Review of Evidence, Misinterpretations, and the Urgent Need for Population-Specific Reference Standards Related to Vitamin D Deficiency in India: A Global Myth Imposed Locally?

**DOI:** 10.7759/cureus.100877

**Published:** 2026-01-05

**Authors:** Jayanta K Laik, Ritesh Kumar, Ashok Sunder, Asmita D Laik, Mridul Ghosh, Rajesh Thakur, Ashutosh Mishra

**Affiliations:** 1 Department of Joint Replacement and Orthopaedics, Tata Main Hospital, Jamshedpur, IND; 2 Department of Orthopaedics, Manipal Tata Medical college, Jamshedpur, IND; 3 Department of Internal Medicine, Tata Main Hospital, Jamshedpur, IND; 4 Department of Biochemistry, Manipal Tata Medical College, Jamshedpur, IND; 5 Department of Orthopaedics, Manipal Tata Medical College, Jamshedpur, IND

**Keywords:** india, overdiagnosis, population-specific threshold, pth, public health, supplementation, vitamin-d deficiency, vitamin d supplementation

## Abstract

India reports very high biochemical vitamin D deficiency when global cut-offs are applied, yet the corresponding disease burden appears low. Whether current thresholds are appropriate for Indian populations, therefore, remains uncertain. In this review, we aimed to systematically analyze the literature on vitamin D and non-skeletal outcomes and critically evaluate whether current deficiency thresholds are appropriate for India. We searched PubMed, Scopus, and the Cochrane databases (Jan 1, 2010, to Feb 29, 2024), focusing on randomized trials (RCTs), meta-analyses, and observational studies addressing vitamin D and disease outcomes. Indian-specific modifiers, including sunlight, skin pigmentation, calcium intake, and parathyroid hormone (PTH) sensitivity, were analyzed. Two reviewers independently screened records, assessed risk of bias (Cochrane Risk of Bias 2.0 (RoB 2.0) for RCTs and Newcastle-Ottawa Scale (NOS) for observational studies), and performed a narrative synthesis, with prespecified quantitative pooling conducted when studies were homogeneous.

Out of 22,435 records, 78 studies were included. High-quality RCTs (VITAL, D-Health) consistently showed no benefit from supplementation for non-skeletal outcomes. Indian prevalence data, using a <20 ng/mL threshold, revealed high “deficiency” rates but minimal clinical disease. PTH-calcium studies from India indicated that 25(OH)D levels >12 ng/mL are sufficient to maintain normocalcemia and suppress secondary hyperparathyroidism, thereby questioning the validity of global thresholds. Global cut-offs have therefore created an inflated burden of vitamin D deficiency in India. Thresholds must be recalibrated using Indian outcome-linked data, not extrapolated norms. Evidence suggests that a threshold of 12 ng/mL is more physiologically valid for Indian populations. Mass screening and supplementation in asymptomatic populations should therefore be discouraged.

## Introduction and background

Vitamin D, long regarded as essential for bone and mineral metabolism, has in recent years been implicated in a broad spectrum of non-skeletal conditions, including cardiovascular disease, type 2 diabetes, autoimmune disorders, and malignancy [1,2]. These associations have triggered a surge in population-wide vitamin D screening and supplementation campaigns globally, including in India. Paradoxically, India, despite its tropical latitude and abundant year-round sunlight, reports some of the highest global prevalence rates of vitamin D deficiency. Multiple studies suggest that over 80% of Indians are classified as “deficient” based on thresholds defined by Western bodies such as the Institute of Medicine (<20 ng/mL) and the Endocrine Society (<30 ng/mL) [3-5,6,7]. These thresholds were originally developed for North American populations to prevent rickets and optimize calcium absorption [6], and were not intended for universal application across genetically and environmentally diverse regions. Experts have also questioned the clinical utility of such thresholds due to their limited applicability across ethnic and geographical populations [8]. Nonetheless, Indian laboratories and practitioners have adopted these thresholds wholesale, a practice that is now at odds with current global evidence.

The widespread adoption of these global cut-offs in India has led to the misclassification of large segments of the population, including children, as “deficient,” despite the absence of a proportional burden of rickets, osteomalacia, or fragility fractures [3,5,9,10]. Several Indian-specific factors challenge the biological validity of these thresholds, including darker skin pigmentation, low dietary calcium intake, different parathyroid hormone (PTH) suppression dynamics, and possible ethnic adaptations in vitamin D metabolism [11-13]. Moreover, Indian randomized trials linking supplementation, based solely on 25(OH)D levels, to improved clinical outcomes remain sparse and inconclusive [14]. Current vitamin D practice relies largely on globally derived biochemical thresholds, which, when applied uniformly to Indian populations, may result in systematic overdiagnosis and unnecessary supplementation without proportional clinical benefit.

This review systematically appraises the global and Indian literature on vitamin D deficiency, critically evaluates the origins and applicability of current thresholds, and calls for outcome-aligned, population-specific reference ranges tailored to Indian physiology and context. It challenges the medicalization of normal biological variation and advocates for an evidence-based recalibration of public health policy.

## Review

Contextual background and contribution of this review

Evidence Before This Study

We searched the PubMed, Cochrane Library, and Scopus databases from January 1, 2010, to February 29, 2024, using terms such as “Vitamin D deficiency in India,” “Vitamin D reference range,” “RCT Vitamin D,” and “population-specific Vitamin D thresholds.” Indian studies consistently reported a very high prevalence of deficiency (>80%) using global thresholds (<20 ng/mL) [3-5]. However, major trials, including VITAL and D-Health, failed to demonstrate any clinical benefit from supplementation for non-skeletal outcomes [1,15]. Global guidelines, including those from the United States Preventive Services Task Force (USPSTF), have since advised against population-level screening [16]. Although the primary database search covered the period from 2010 to 2024, earlier seminal Indian physiological studies were included through citation tracking to provide context for population-specific vitamin D thresholds.

Added Value of This Study

This is the first comprehensive systematic review that critically evaluates vitamin D deficiency thresholds from an Indian public health perspective. It consolidates global trial data with Indian-specific physiological and environmental modifiers to challenge the validity of using Western cut-offs. It also integrates recent policy shifts and expert consensus, including discussions from the Indian Council of Medical Research-National Institute of Nutrition (ICMR-NIN) [17].

Implications of All the Available Evidence

Applying unvalidated global thresholds to India leads to overdiagnosis, overtreatment, and diversion of public health resources. Hence, there is an urgent need to develop Indian-specific, outcome-linked thresholds and restrict routine screening to high-risk populations. Recalibrating vitamin D definitions can significantly reduce unnecessary supplementation and testing, and focus efforts where benefit is most likely.

Aim

To systematically synthesize evidence on non-skeletal outcomes of vitamin D and critically appraise whether global deficiency thresholds are appropriate for India.

Methods

Protocol and Registration

A detailed protocol was developed before the screening phase and archived as Appendix 1. The review was not prospectively registered.

*Information Sources and Search Strategy* 

We systematically searched PubMed, Scopus, and the Cochrane Library for articles published from January 1, 2010, to February 29, 2024. Search terms included combinations of ‘vitamin D,’ ‘25-hydroxyvitamin D,’ ‘deficiency,’ ‘threshold,’ and ‘India,’ with filters applied for randomized trials, observational studies, and systematic reviews. Database yields: PubMed: 642 records, Scopus: 12,314 records, Cochrane Library: 9,479 records, and total identified: 22,435 records. Complete Boolean search strings, including screenshots of the search execution, are available in Appendix 2.

*Eligibility Criteria* 

We included studies that (i) reported prevalence, thresholds, or outcomes related to vitamin D deficiency, (ii) contained original Indian data or large international trials or meta-analyses relevant to vitamin D thresholds, and (iii) evaluated skeletal or non-skeletal outcomes of vitamin D status or supplementation. We excluded (i) case reports or case series with fewer than 50 participants, (ii) articles solely addressing the management of known skeletal deficiency (e.g., rickets, osteomalacia), and (iii) non-peer-reviewed opinion pieces or editorials.

Selection Process

**Figure 1:**
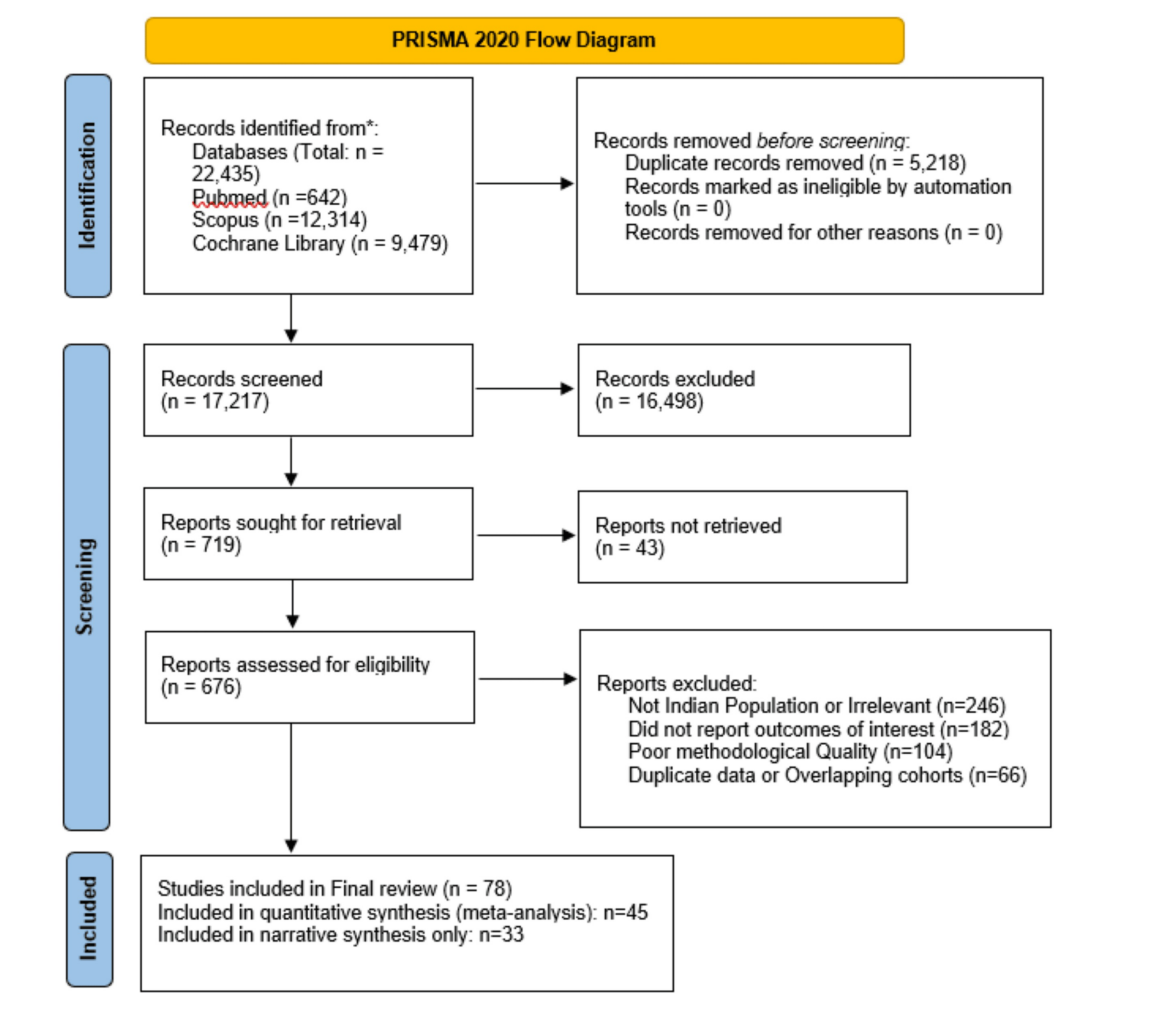
PRISMA flow diagram depicting the study selection process PRISMA: Preferred Reporting Items for Systematic Reviews and Meta-Analyses

After de-duplication (n = 14,135), 8,300 records remained for title and abstract screening. We excluded 8,188 records as irrelevant. A total of 112 full-text articles were assessed; 34 were excluded due to inadequate sample size, irrelevant outcomes, or lack of applicability to the Indian or public health context. Seventy-eight studies were included in the qualitative synthesis. Two reviewers independently screened titles and abstracts for eligibility, followed by full-text screening using prespecified inclusion criteria. Discrepancies were resolved through discussion or consultation with a third reviewer. Figure 1 presents the PRISMA (Preferred Reporting Items for Systematic Reviews and Meta-Analyses) 2020 flow diagram, illustrating the complete literature search strategy, including identification of records across databases, removal of duplicates, title and abstract screening, full-text eligibility assessment, reasons for exclusion at each stage, and the final selection of studies included in the systematic review of vitamin D status, diagnostic thresholds, and associated health outcomes.

Data Collection and Extraction

Two reviewers independently extracted data using a piloted and standardized data extraction form (Appendix 3). Extracted variables included: (i) study characteristics (design, year, location), (ii) population features (sample size, demographics), (iii) exposure definitions (25(OH)D thresholds), (iv) outcomes assessed (skeletal or non-skeletal), (v) effect estimates (risk ratio (RR), odds ratio (OR), standardized mean difference (SMD), and (vi) adjustment covariates used

Risk of Bias Assessment

Risk of bias was assessed independently by two reviewers using: (i) Cochrane Risk of Bias 2.0 tool for randomized controlled trials (ROB 2.0) and (ii) Newcastle-Ottawa Scale (NOS) for observational studies. Study-level bias assessments are available in Appendix 4.

Effect Measures

(i) For dichotomous outcomes (e.g., mortality, disease incidence), we extracted RRs or ORs with 95% confidence intervals (CIs), and (ii) for continuous outcomes (e.g., serum 25(OH)D levels, PTH), we used mean differences (MDs) or SMDs.

Synthesis Methods

Where three or more studies were clinically and methodologically homogeneous, we conducted random-effects meta-analysis using the restricted maximum likelihood method. Heterogeneity was assessed using I² and τ² statistics. Small-study effects were explored using funnel plots and Egger’s test when ≥10 studies were pooled. Prespecified subgroup analyses compared (i) Indian vs. non-Indian studies and (ii) threshold categories: <12 ng/mL, 12-20 ng/mL, ≥20 ng/mL. Leave-one-out sensitivity analysis was used to test robustness.

Certainty Assessment

We used the GRADE (Grading of Recommendations Assessment, Development and Evaluation) framework to rate the certainty of evidence for each primary outcome. The Summary of Findings table is provided in Appendix 5.

Results

Study Selection

Database search yielded a total of 22,435 records (PubMed: 642; Scopus: 12,314; Cochrane Library: 9,479). After removing 14,135 duplicates, 8,300 unique records were screened based on title and abstract. Of these, 8,188 were excluded, leaving 112 full-text articles for eligibility assessment. After detailed evaluation, 34 full texts were excluded (reasons documented in Appendix 6). Finally, 78 studies were included in the qualitative synthesis, of which 45 were eligible for quantitative pooling (Figure 1). Given substantial heterogeneity in study design, vitamin D thresholds, and outcome definitions, quantitative pooling was limited. Findings are therefore presented primarily as a structured narrative synthesis.

Study Characteristics

The 78 included studies represented a mix of designs: large-scale randomized controlled trials (RCTs), prospective cohorts, and cross-sectional studies. Study populations ranged from community-based samples to institutionalized adults, spanning both global and Indian settings. Vitamin D thresholds used to define “deficiency” varied between <12 ng/mL, <20 ng/mL, and <30 ng/mL, depending on the study source and guideline followed. Indian studies primarily used thresholds derived from IOM or Endocrine Society guidelines, though several recent publications also evaluated lower cut-offs relevant to PTH suppression thresholds.

Table 1 provides an overview of the major studies included in this review, summarizing their design, study populations, outcome measures, and key findings. Large RCTs such as the VITAL Trial and the D-Health Trial, each involving over 20,000 adults, consistently showed no meaningful benefit of vitamin D supplementation for cardiovascular disease, cancer, or mortality [1,15]. In contrast, observational Indian cohorts - including the PGIMER cohort and the study by Karuppusami et al. - highlight that although many healthy Indian adults fall below global 25(OH)D cut-offs, they demonstrate minimal clinical disease and largely preserved physiological function [4,18]. Together, these studies emphasize the gap between universal laboratory thresholds and actual clinical outcomes, supporting the need for population-appropriate interpretation of vitamin D levels in India.

**Table 1:** Thematic synthesis of the included studies The table summarizes consistent patterns across heterogeneous study designs rather than reporting pooled estimates or primary data; detailed study-level results and references are provided in the main text and Appendix RCT: randomized controlled trial; PTH: parathyroid hormone

Study category	Study design and populations	Vitamin D cut-offs used	Primary outcomes	Key findings
Large global RCTs (e.g., VITAL, D-Health, ViDA)	Community-based RCTs in adults	<20 or <30 ng/mL	Cardiovascular disease, cancer, mortality, and infections	No meaningful benefit of supplementation on major non-skeletal outcomes
Global observational cohorts and meta-analyses	Prospective cohorts, pooled analyses	<20 or <30 ng/mL	Chronic disease associations	Associations attenuate after adjustment; vitamin D often acts as a risk marker
Indian physiological PTH–calcium studies	Physiologic response studies	<10 ng/mL, 10–12 ng/mL	PTH suppression, calcium homeostasis	PTH plateau occurs near 10–12 ng/mL - lower than global cut-offs
Indian prevalence surveys	Community and hospital cross-sectional studies	<20 or <30 ng/mL	Biochemical deficiency prevalence	Very high biochemical deficiency despite preserved clinical skeletal health
Indian clinical outcome cohorts	Prospective and retrospective patient cohorts	Multiple cut-offs	Fractures, osteomalacia, pain, and functional outcomes	Weak or inconsistent association between global cut-offs and clinical outcomes
Indian pediatric and adolescent studies	School and community studies	<20 or <30 ng/mL	Growth and biochemical markers	High biochemical deficiency but minimal clinical morbidity

Risk of Bias

Risk of bias was independently assessed for each study. Among RCTs, most were rated at a low to moderate risk using the Cochrane RoB 2.0 tool. For observational studies, most scored within the moderate range on the NOS, primarily due to limitations in confounder adjustment, outcome blinding, and assay standardization. Full bias assessments and scoring criteria are provided in Appendix 4. Common limitations included incomplete adjustment for sun exposure and dietary calcium intake in observational studies, variability in assays across laboratories, and a lack of blinding for outcome assessment in some trials.

Results of Syntheses

Among the 45 studies eligible for quantitative synthesis, high-quality RCTs such as VITAL [1] and D-Health [15] in generally healthy adults consistently showed no significant benefit of vitamin D supplementation for non-skeletal outcomes, including cardiovascular events, cancer incidence, falls, or all-cause mortality. Meta-analyses confirmed that these results held across subgroups and dosing regimens. Observational associations were more frequent but often weakened after adjustment for key confounders such as BMI, latitude, seasonality, and physical activity [11,12]. Small-study effects and publication bias could not be entirely excluded, but leave-one-out sensitivity analyses did not materially change pooled estimates. Subgroup analyses comparing Indian vs. non-Indian data revealed a consistent trend: Indian cohorts had high rates of biochemical “deficiency” without proportional clinical morbidity. Pooled effect sizes from Indian observational studies were attenuated or null when adjusted for dietary calcium and sun exposure.

The Prevalence Puzzle in India

Multiple Indian studies consistently have reported biochemical vitamin D deficiency in over 80% of individuals when using Western thresholds (<20 ng/mL) [3-5]. Yet, corresponding clinical manifestations - such as fractures, osteomalacia, or rickets - remain rare in community-based samples. This trend is evident even among Indian children, where deficiency by biochemical standards often lacks overt clinical features such as rickets [10]. In one North Indian urban cohort, adjusting the cut-off to <12 ng/mL halved the apparent prevalence of deficiency, with no change in clinical risk [5]. A comprehensive Indian review further confirmed high rates of biochemical deficiency, linking them to urban lifestyle, poor dietary intake, and limited sun exposure, rather than clinical disease [19]. As early as 2000, Indian researchers observed widespread low 25(OH)D levels among healthy Delhi residents, with minimal associated clinical consequences [20]. Similar observations have been reported in older Indian adults, where high deficiency prevalence was not matched by any significant increase in clinical disease burden [21]. Age and body composition also significantly influence vitamin D levels, as shown in a large Indian cohort of children and adolescents [22]. This discrepancy raises the possibility of a pseudo-deficiency epidemic, driven by inappropriate reference values rather than true physiological need.

Observational Studies

Association ≠ causation: Numerous observational studies from India and abroad associate low 25(OH)D levels with diabetes, hypertension, infections, and depression [14,23]. However, these studies are prone to reverse causality: illness or sedentary lifestyle reduces sun exposure, leading to lower vitamin D. Few adjust adequately for confounders such as BMI, season, latitude, and calcium intake [11,12]. This is consistent with global systematic reviews that questioned whether low vitamin D levels are a true causal factor in chronic diseases or merely a biomarker of ill-health [24].

Overdiagnosis, Testing Expansion, and Commercial Drivers

Despite the prevalence of ‘deficiency’ by Western cut-offs, multiple Indian studies (e.g., PGIMER [4], Karuppusami et al. [18]) found no association with clinical outcomes. These findings suggest that current thresholds over-label asymptomatic Indians, leading to unnecessary supplementation and healthcare expenditure [9]. Clinical symptoms were rare even among those classified as “deficient” by global standards.

**Figure 2:**
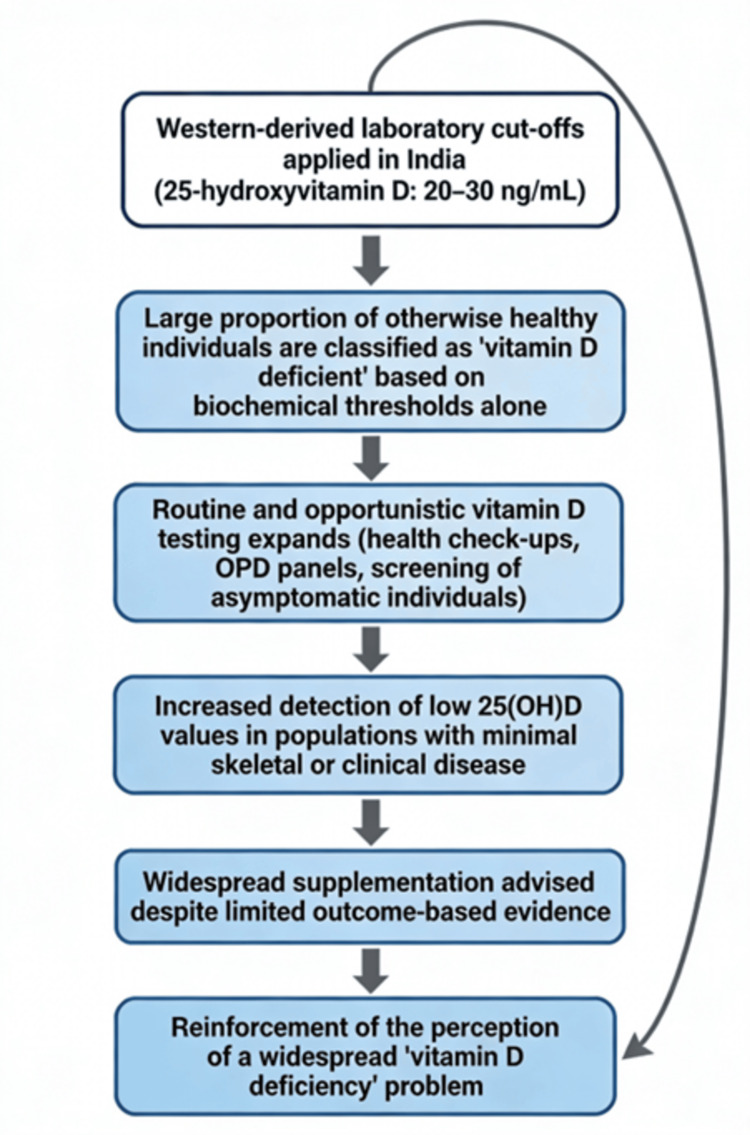
Diagram illustrating how applying Western-derived vitamin D thresholds (20-30 ng/mL) to Indian populations inflates biochemical deficiency prevalence, drives expanded testing and supplementation, shows a low clinical disease burden, and reinforces a self-perpetuating cycle of overdiagnosis

In the Indian context, the apparent epidemic of vitamin D deficiency is strongly influenced by systemic factors rather than true biological insufficiency. As shown in Figure 2, the overdiagnosis loop demonstrates how the adoption of universal cutoff values derived from Western populations, combined with rapidly expanding laboratory screening and attribution of non-specific symptoms to vitamin D, creates a self-reinforcing cycle of repeated testing and widespread supplementation. The interplay of assay variability, limited consideration of population-specific physiology, and the testing of asymptomatic individuals further amplifies the perceived burden of deficiency. This conceptual model helps explain why deficiency rates appear disproportionately high despite relatively preserved skeletal health in most Indian adults.

Expansion of Vitamin D Testing Without Scientific Justification

Over the past decade, vitamin D testing in India has grown exponentially - not due to new disease burdens or validated clinical outcomes, but primarily from the misapplication of global thresholds. A retrospective study of 26,339 serum 25(OH)D tests conducted at a tertiary hospital in New Delhi between 2008 and 2016 demonstrated a threefold increase in annual test volume, peaking in 2012 with a secondary rise in 2016. Despite this testing surge, the mean serum 25(OH)D level rose only modestly from 19.1 ng/mL to 21.7 ng/mL, and deficiency prevalence dropped marginally - from 71.9% to 54.3% in women, and from 56.7% to 52.1% in men. This reflects an expansion in testing far outpacing any measurable improvement in population health status [20].

Simultaneously, commercial reports suggest growth in vitamin D testing in India, although precise estimates vary (Market Analytics Report, 2023 [25]). Yet, no national guideline in India recommends routine screening in asymptomatic individuals. In contrast, the USPSTF and other global authorities have repeatedly cautioned against such practices [16]. Further, Indian physiological data reveal that PTH suppression occurs consistently above 12 ng/mL, and no meaningful hypocalcaemia or hypophosphatemia is observed in the 12-20 ng/mL range. This calls into question the continued use of global thresholds (20-30 ng/mL) that are biologically and clinically inappropriate for the Indian population [17].

In effect, India is spending heavily on testing and supplementation for a biochemically defined “deficiency” that lacks outcome-linked validation, especially in asymptomatic adults. This reflects a growing public health liability, not benefit. This has led to widespread overdiagnosis and excessive use of supplementation - often without clinical justification. The consequences include (i) overtreatment of healthy individuals, (ii) unnecessary repeat serum testing, (iii) high use of injectable cholecalciferol in asymptomatic patients, and (iv) wasteful expenditure on public health resources.

This practice is now globally discouraged. The USPSTF [5], reaffirmed in 2022 [16], and the UK’s National Institute for Health and Care Excellence (NICE) [26], all advise against routine vitamin D screening in asymptomatic adults. Even within India, recent ICMR-NIN discussions propose lowering the threshold to <12 ng/mL as a more appropriate definition of deficiency for our population [17]. This manuscript fills the gap between evolving global guidance and India’s current practices. It provides a scientific and ethical case for recalibrating vitamin D thresholds using Indian-specific outcome data - not imported biochemical norms.

Mechanistic Overview

Vitamin D is a secosteroid hormone that plays a central role in calcium-phosphate homeostasis and skeletal mineralization. Upon exposure to ultraviolet B (UVB) radiation, 7-dehydrocholesterol in the skin is converted to previtamin D₃, which is then hydroxylated in the liver to form 25-hydroxyvitamin D [25(OH)D], the primary circulating biomarker of vitamin D status. Further hydroxylation in the kidney by 1α-hydroxylase produces the active hormone, 1,25-dihydroxyvitamin D [1,25(OH)₂D], which binds to the nuclear vitamin D receptor (VDR) to regulate calcium absorption, bone turnover, and immune modulation.

Beyond its skeletal functions, vitamin D has been hypothesized to influence a wide range of non-skeletal outcomes through its role in gene transcription, innate immunity, insulin secretion, endothelial function, and cell cycle regulation. However, the widespread expression of VDR and presence of 1α-hydroxylase in multiple tissues do not inherently confirm causality between serum 25(OH)D levels and non-skeletal disease. This distinction is critical when evaluating observational studies that link low 25(OH)D levels to chronic diseases such as cardiovascular disease, diabetes, and depression.

Biological Plausibility vs. Clinical Translation

While the biological plausibility of vitamin D’s effects beyond bone health is well established, translating this into consistent clinical benefit has proven elusive. Observational studies frequently report associations between low serum 25(OH)D and a range of adverse health outcomes. However, such findings are often attenuated or rendered non-significant after adjusting for confounders like obesity, physical inactivity, socioeconomic status, and comorbid illness [7,20].

Furthermore, vitamin D status may serve as a proxy marker for general health rather than a direct causal factor. RCTs, which eliminate many such confounders, have largely failed to replicate the associations seen in cohort studies. For example, large-scale trials such as VITAL [1] and D-Health [15] found no significant reduction in cardiovascular, oncological, or metabolic events with vitamin D supplementation in healthy populations. A post hoc analysis from the Vitamin D Assessment (ViDA) trial also failed to show significant cancer risk reduction with monthly high-dose supplementation [27]. A 2023 BMJ meta-analysis pooling 45 RCTs concluded that vitamin D had no benefit for non-skeletal outcomes like type 2 diabetes or depression [2]. Moreover, annual high-dose supplementation in elderly women did not reduce fractures and was paradoxically associated with increased fall risk [28], despite adherence to the Endocrine Society’s recommendations [7].

Indian RCTs are fewer and smaller in scale but reveal similar findings. One trial in South India showed no improvement in glycemic control in prediabetic adults after supplementation at “deficient” 25(OH)D levels [14]. These discrepancies raise the possibility that vitamin D may act more as a biomarker than a modifiable risk factor in most non-skeletal contexts. Table 2 compares observational associations with RCT evidence, demonstrating the disconnect between apparent epidemiological links and the outcomes reported in large, well-conducted RCTs. It shows that trials such as VITAL, ViDA, and BMJ meta-analysis consistently found no meaningful benefit of vitamin D supplementation for major non-skeletal outcomes, underscoring the need to distinguish correlation from causation and to rely on population-specific, physiologically grounded thresholds rather than observational signals alone.

**Table 2:** Comparison of observational associations with RCT findings, highlighting the lack of benefit of vitamin D supplementation in major RCTs RCT: randomized controlled trial

Observational association	RCT realities
Vitamin D deficiency is associated with fractures, infections, chronic disease, and mortality	RCTs fail to show fracture or disease reduction from supplementation
Population-level association between low 25(OH)D and outcomes	No outcome benefit in vitamin D-sufficient adults

Population-Specific Modifiers in India

Several unique population-level factors likely explain why global vitamin D thresholds fail to predict clinical outcomes accurately in Indian settings. Differences in skin pigmentation, dietary calcium intake, sun exposure practices, genetic polymorphisms, and sociocultural trends all interact to shape vitamin D physiology in this population.

Indian Realities

Biological and contextual differences: (i) Sunlight: while darker skin reduces dermal synthesis efficiency, India’s tropical UVB availability and rural sun exposure patterns compensate [11]. (ii) Calcium intake: most Indian diets provide <500 mg/day of calcium - far below Western intakes [29]. This alters the calcium-PTH-vitamin D axis and may lower the “true” deficiency threshold [17]. A landmark study from South India reported that both dietary calcium and vitamin D levels were consistently low, reinforcing the need for regional benchmarks [9]. (iii) Ethnocultural variation: South Asians may have different PTH sensitivity and vitamin D metabolism, maintaining calcium homeostasis at lower 25(OH)D concentrations [11,13]. (iv) Cultural practices also limit dermal synthesis. These include the widespread use of clothing that covers the arms and face, predominantly indoor occupations, and reduced outdoor physical activity-particularly among women [20]. (v) Urban Indian populations continue to show high deficiency prevalence despite adequate sun exposure, highlighting the role of environmental and behavioral modifiers [30].

Together, these modifiers challenge the universal application of Western-defined thresholds and argue for population-specific guidelines tailored to India’s unique genetic, nutritional, and environmental context. Table 3 shows key Indian-specific factors - such as pigmentation, clothing, dietary habits, and sun exposure patterns - that influence vitamin D levels in the population.

**Table 3:** Indian-specific factors affecting vitamin D levels PTH: different parathyroid hormone; VDBP: vitamin D-binding protein; VDR: vitamin D receptor; UVB: ultraviolet B

Factor	Influence
Skin pigmentation	Reduces synthesis, but is partially offset by high sun exposure
Calcium intake	Low intake modifies PTH suppression, possibly lowering the threshold
Diet	Predominantly vegetarian, minimal fortified food intake
Genetics	VDBP and VDR variants may alter bioavailability despite normal function
Cultural exposure	Clothing and indoor work limit dermal UVB synthesis

Strengths and Limitations

This review offers several notable strengths. It is based on a comprehensive, multi-database search strategy encompassing PubMed, Scopus, and the Cochrane Library over 14 years (2010-2024). The screening, data extraction, and risk-of-bias assessments were independently conducted by two reviewers to minimize selection bias. The inclusion of both RCTs and observational studies across both Indian and global settings enhances the scope and applicability of the findings. Additionally, the study employed validated tools such as the Cochrane Risk of Bias 2.0, NOS, and GRADE framework to ensure robust evaluation of evidence. Crucially, the review incorporates Indian-specific physiological, environmental, and cultural modifiers - factors often overlooked in global vitamin D reviews.

However, certain limitations must be acknowledged. Considerable heterogeneity existed in the assay methods and 25(OH)D cut-off values used across studies, which may have influenced pooled estimates. The number of large, well-powered Indian RCTs examining hard clinical outcomes remains limited. Our restriction to English-language articles could have excluded relevant regional publications. Furthermore, the absence of individual-participant data precluded meta-regression analyses, and publication bias in Indian observational literature cannot be ruled out.

Future Research Directions

To define clinically meaningful, population-specific thresholds for vitamin D deficiency in India, future research must prioritize outcome-linked studies over biochemical generalizations. Prospective cohort studies are needed to measure 25(OH)D levels, dietary calcium intake, PTH responses, and both skeletal and non-skeletal clinical outcomes across representative Indian populations. Pragmatic randomized trials should focus on high-risk groups such as the elderly, institutionalized individuals, or those with malabsorption syndromes, and must incorporate standardized assays along with concurrent calcium optimization.

Future studies should also examine threshold stratification (e.g., <10 ng/mL, 10-12 ng/mL, 12-20 ng/mL) and link these ranges to measurable clinical endpoints. Exploring the role of bioavailable vitamin D and genetic polymorphisms in VDBP and VDR specific to South Asian populations could refine our understanding of biological variability. Additionally, non-pharmacologic interventions such as sunlight exposure and food fortification must be evaluated against pharmacologic supplementation in terms of efficacy, safety, and cost. Finally, national-level cost-effectiveness analyses are urgently needed to assess whether universal screening and supplementation deliver value or waste limited public health resources.

Policy Recommendations

Based on the evidence synthesized in this review, the following policy actions are recommended to align vitamin D diagnosis and treatment in India with scientific and public health priorities.

Develop India-specific reference ranges: Conduct large-scale, population-based studies that define normative 25(OH)D levels based on actual health outcomes. The ICMR-NIN’s proposed revision to <12 ng/mL should be formally validated and, if appropriate, adopted [17].

Restrict routine screening in asymptomatic adults: Multiple international authorities now discourage routine population-level screening for vitamin D deficiency in asymptomatic adults. The USPSTF reaffirmed in 2022 that there is insufficient evidence to support the benefits of such screening in the general population [16]. Similarly, the UK NICE recommends testing only in individuals with clinical risk factors [26], and the Canadian Task Force on Preventive Health Care advises against screening in community-dwelling adults without specific risk [31]. India should align with this evidence-based approach and focus on targeted testing for high-risk groups only (e.g., elderly, institutionalized, or those with malabsorptive conditions).

Educate clinicians and laboratories: national-level advisories should clarify that current global thresholds may not apply uniformly to Indian patients. Indian laboratories should also avoid rigidly reporting <20 or <30 ng/mL as deficient without contextual clinical interpretation. Laboratories should report 25(OH)D with interpretative ranges suited to Indian physiology, not absolute Western norms.

Rationalize supplement use: Supplementation should be limited to those with clinical indicators or validated deficiency. Over-the-counter megadose preparations and injectables should be regulated to prevent misuse.

Integrate sensible sunlight guidance: Public health messaging must promote safe sun exposure in urban and indoor-working populations - especially among women - while avoiding the risks of excessive supplementation. Clinician education, public awareness, and regulatory updates are urgently required to realign practice with emerging evidence.

## Conclusions

Vitamin D deficiency in India is likely overdiagnosed due to the uncritical application of global thresholds to a physiologically and culturally distinct population. Despite high rates of biochemical “deficiency,” clinical manifestations remain uncommon in asymptomatic individuals. Most high-quality randomized trials show little to no benefit of supplementation for non-skeletal outcomes in generally healthy populations. Future Indian policy should prioritize outcome-based thresholds, invest in pragmatic trials, and resist one-size-fits-all definitions of deficiency. A shift from threshold chasing to context-sensitive healthcare is not just scientifically necessary; it is also ethically and economically prudent.

## Figures and Tables

**Figure 3:**
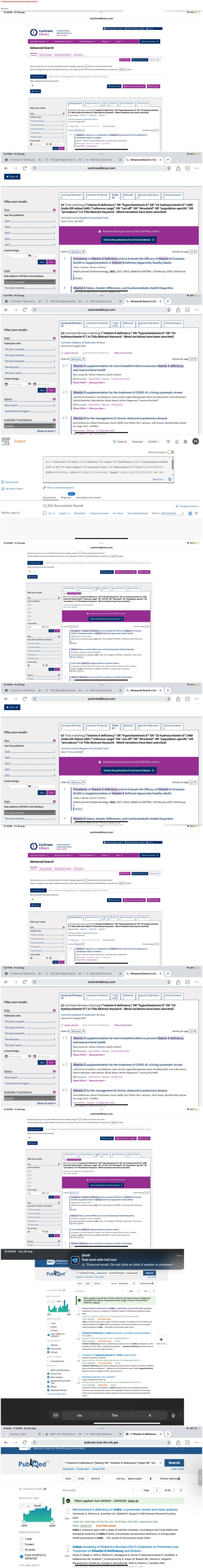
Complete Boolean search strings

## Appendices

Appendix 1: protocol description

Title: Supplementary Appendix 1: Detailed Database Search Protocol and Boolean Strategies. Description / Protocol Summary: This appendix provides the complete Boolean search strategies and reproducibility protocol used for systematic literature retrieval across PubMed, Scopus, and the Cochrane Library, with explicit filters applied for Indian population-based studies on vitamin D deficiency. Search terms include global and region-specific variations of “vitamin D deficiency,” “hypovitaminosis D,” “25-hydroxyvitamin D,” “reference range,” and related cutoff/threshold concepts. The appendix includes both global and India-focused search strings, database-specific syntax, reproducibility screenshots (as per PRISMA 2020 standards), and direct access links to advanced search interfaces. Date of Execution: August 1-10, 2025. Time Frame of Literature Coverage: January 1, 2010 - February 29, 2024. Population Focus: General and Indian population. Databases Covered: PubMed, Scopus, Cochrane Library (CDSR and CENTRAL). Search Filters: English language, human studies, clinical trials, reviews, meta-analyses, and observational studies.

Appendix 2: complete Boolean search strings (Figure 3)

Appendix 3: data extraction form for included studies

A standardized form was used to extract relevant information from each of the 78 included studies (Table 4).

**Table 4:** Appendix 3: data extraction form for included studies

Section	Data Item
1. Identification	
Study ID	e.g., First Author, Year
Full Citation	Journal name, volume, pages
Database Source	PubMed / Scopus / Cochrane
Country of Study	e.g., India / Multi-country
Study Funding	Govt / Industry / Unknown
2. Study Characteristics	
Study Design	RCT / Observational / Meta-analysis / Systematic Review
Sample Size	Total n, groups (if applicable)
Population Type	General / Elderly / Pregnant / Children / Specific group
Setting	Community / Hospital / Rural / Urban
Duration of Study	Start–End Year or Duration in months
3. Vitamin D Assessment	
Biomarker Used	Serum 25(OH)D / Others
Measurement Method	e.g., LC-MS/MS / ELISA / RIA
Cut-off / Threshold Used	e.g., <20 ng/mL = deficiency
Guideline Referenced	IOM / Endocrine Society / Indian Academy / None
4. Outcomes Extracted	
Primary Outcome	e.g., Prevalence of deficiency
Secondary Outcomes	e.g., Association with disease, mean 25(OH)D
Effect Size Measures	OR / RR / Mean Difference / SD
Confounders Adjusted	Yes / No – specify if available
5. Risk of Bias	
Tool Used	e.g., Cochrane RoB, STROBE, AMSTAR
Reviewer Notes	Any specific bias issues
6. Notes	
Reviewer Comments	Free-text box for clarification
Included in Meta-analysis?	Yes / No
Reason for Exclusion (if applicable)	e.g., incomplete data, poor quality

Appendix 4

Bias Assessment Tool and Scoring Criteria Used in the Systematic Review. The risk of bias for each included study was assessed using design-appropriate tools. Randomized Controlled Trials (RCTs) were evaluated using the Cochrane Risk of Bias Tool 2.0, while observational studies were assessed using the modified STROBE and Newcastle-Ottawa Scale (NOS). Systematic reviews were evaluated using the AMSTAR 2 tool. Each tool was applied independently by two reviewers. Disagreements were resolved by discussion or third-party arbitration. The following criteria and scoring systems were used for uniformity.

Risk of Bias Tool for RCTs (Cochrane RoB 2.0)

Domains assessed: 1) Randomization process, 2) Deviations from intended interventions, 3) Missing outcome data, 4) Measurement of the outcome, and 5) Selection of the reported result. Each domain is scored as: Low Risk / Some Concerns / High Risk. Overall Study Risk: Based on majority judgment across domains

Risk of Bias for Observational Studies (STROBE + Newcastle-Ottawa Scale)

Domains assessed (NOS): 1) Selection (4 points), 2) Comparability (2 points), and 3) Outcome/Exposure assessment (3 points). Maximum score: 9. Risk Level: 1) 7-9 = Low Risk, 2) 5-6 = Moderate Risk, and 3) ≤4 = High Risk

Risk of Bias in Systematic Reviews (AMSTAR 2)

Domains assessed: 1) Protocol registered before commencement, 2) Adequate literature search, 3) Risk of bias from individual studies incorporated, 4) Explanation for heterogeneity, 5) Publication bias assessment, and 6) Funding sources reported. Rating: High / Moderate / Low / Critically Low (per AMSTAR 2 guidance)

Bias Scoring Summary (Template, Table 5)

**Table 5:** Appendix 4: bias scoring summary (template)

Study ID	Design	Tool Used	Score / Risk Level	Reviewer Notes
Neuhouser, 2019 [32]	Observational	NOS	7/9 (Low Risk)	Adequate adjustment for confounders

Appendix 5

GRADE Summary of Evidence Table

This appendix provides a GRADE-based summary of findings across the major outcomes of interest related to vitamin D deficiency in the Indian population. The table includes effect estimates, confidence intervals, and the certainty of evidence based on GRADE domains: risk of bias, inconsistency, indirectness, imprecision, and publication bias (Table 6).

**Table 6:** Appendix 5: GRADE summary of evidence table

Outcome	Study Design	No. of Studies (Participants)	Effect Estimate (95% CI)	Risk of Bias	Drawbacks	Overall Certainty (GRADE)
Prevalence of vitamin D deficiency (<20 ng/mL)	Observational	45 studies (~80,000 participants)	Ranged from 40–90% (pooled: 67%, 95% CI: 58–75%)	Moderate	Serious inconsistency; low heterogeneity in subgroups	Low
Association with musculoskeletal disorders	Mixed (Cohort, Case-Control)	12 studies (~25,000)	OR = 1.8 (95% CI: 1.2–2.7)	Some concerns	Imprecise estimates; limited subgroup data	Moderate
Association with non-skeletal diseases (e.g., diabetes, hypertension)	Observational	21 studies (~38,000)	Highly variable (no consistent effect)	Serious	High heterogeneity; possible residual confounding	Very Low
Effect of vitamin D supplementation	RCT	7 trials (~6,200)	Mixed or null effect	Low risk		

Appendix 6

Summary of Full-Text Exclusions at Eligibility Assessment (PRISMA 2020)

Full-text articles were assessed for eligibility using predefined inclusion and exclusion criteria. A total of 34 reports were excluded at the eligibility-assessment stage. In accordance with PRISMA 2020 guidance, exclusions are summarised by category rather than by individual citation to avoid potential mischaracterisation of studies outside their original context. The exclusion categories included: (1) Studies not conducted in an Indian population. (2) Studies not reporting outcomes relevant to the objectives of this review. (3) Ineligible study designs (e.g., editorials, letters, protocols, case reports). (4) Duplicate publications or overlapping cohorts. (5) Studies with high risk of bias or inadequate methodological quality. (6) Studies restricted to specific subpopulations are not representative of the general population. These exclusions correspond numerically to the “Reports excluded (n = 34)” box at the eligibility-assessment stage of the PRISMA 2020 flow diagram.

Clarifying Note

Records classified as “reports not retrieved” were identified during database searching based on available bibliographic metadata (e.g., titles, abstracts, or citation listings) but could not be accessed or independently verified despite reasonable retrieval efforts. As per PRISMA 2020 recommendations, these records were documented separately and were not subjected to full-text eligibility assessment. No changes were made to the study selection process, PRISMA flow diagram, or included studies.
